# Traffic-Related Air Pollution, Oxidative Stress Genes, and Asthma (ECHRS)

**DOI:** 10.1289/ehp.0900589

**Published:** 2009-07-23

**Authors:** Francesc Castro-Giner, Nino Künzli, Bénédicte Jacquemin, Bertil Forsberg, Rafael de Cid, Jordi Sunyer, Deborah Jarvis, David Briggs, Danielle Vienneau, Dan Norback, Juan R. González, Stefano Guerra, Christer Janson, Josep-Maria Antó, Matthias Wjst, Joachim Heinrich, Xavier Estivill, Manolis Kogevinas

**Affiliations:** 1 Centre for Research in Environmental Epidemiology, Barcelona, Spain; 2 Municipal Institute of Medical Research, Hospital del Mar, Barcelona, Spain; 3 CIBER Epidemiología y Salud Pública, Barcelona, Spain; 4 Institució Catalana de Recerca i Estudis Avançats (ICREA), Barcelona, Spain; 5 Institut national de la santé et de la recherche médicale, U780, Epidemiology and Biostatistics, Villejuif, France; 6 Occupational and Environmental Medicine, Department of Public Health and Clinical Medicine, Umeå University, Umeå, Sweden; 7 Genes and Disease Program, Center for Genomic Regulation, Barcelona, Spain; 8 Department of Health and Experimental Sciences, University Pompeu Fabra, Barcelona, Spain; 9 Respiratory Epidemiology and Public Health Group, National Heart and Lung Institute and; 10 Epidemiology and Public Health, Imperial College, London, United Kingdom; 11 Department of Medical Sciences, Uppsala University and University Hospital, Uppsala, Sweden; 12 Department of Medical Sciences, Respiratory Medicine and Allergology, Uppsala University, Uppsala, Sweden; 13 Helmholtz Zentrum München, German Research Centre for Environmental Health, Munich, Germany; 14 Institute of Epidemiology, Helmholtz Zentrum München, Munich, Germany; 15 National School of Public Health, Athens, Greece

**Keywords:** air pollution, asthma, gene polymorphisms, genetics, nitrogen dioxide, oxidative stress, traffic pollution

## Abstract

**Background:**

Traffic-related air pollution is related with asthma, and this association may be modified by genetic factors.

**Objectives:**

We investigated the role of genetic polymorphisms potentially modifying the association between home outdoor levels of modeled nitrogen dioxide and asthma.

**Methods:**

Adults from 13 cities of the second European Community Respiratory Health Survey (ECRHS II) were included (*n* = 2,920), for whom both DNA and outdoor NO_2_ estimates were available. Home addresses were geocoded and linked to modeled outdoor NO_2_ estimates, as a marker of local traffic-related pollution. We examined asthma prevalence and evaluated polymorphisms in genes involved in oxidative stress pathways [gluthatione *S*-transferases M1 (*GSTM1*), T1 (*GSTT1*), and P1 (*GSTP1*) and NAD(P)H:quinine oxidoreductase (*NQO1*)], inflammatory response [tumor necrosis factor α (*TNFA*)], immunologic response [Toll-like receptor 4 (*TLR4*)], and airway reactivity [adrenergic receptor β2 (*ADRB2*)].

**Results:**

The association between modeled NO_2_ and asthma prevalence was significant for carriers of the most common genotypes of *NQO1* rs2917666 [odds ratio (OR) = 1.54; 95% confidence interval (CI), 1.10–2.24], *TNFA* rs2844484 (OR = 2.02; 95% CI, 1.30–3.27). For new-onset asthma, the effect of NO_2_ was significant for the most common genotype of *NQO1* rs2917666 (OR = 1.52; 95% CI, 1.09–2.16). A significant interaction was found between *NQO1* rs2917666 and NO_2_ for asthma prevalence (*p* = 0.02) and new-onset asthma (*p* = 0.04).

**Conclusions:**

Genetic polymorphisms in the *NQO1* gene are related to asthma susceptibility among persons exposed to local traffic-related air pollution. This points to the importance of antioxidant pathways in the protection against the effects of air pollution on asthma.

Asthma is a complex disease with both genetic and environmental components. The interaction between genetic predisposition and environmental factors is likely to have an important role in the etiology and prognosis of the disease. However, only few studies have addressed gene–environment interactions in asthma (Castro-Giner et al. 2006; London 2007; Yang et al. 2008).

Air pollution contributes to the development of asthma (Brauer et al. 2007; Gehring et al. 2002; Jacquemin et al. 2009; McConnell et al. 2006; Modig et al. 2006) and asthma exacerbations (D’Amato et al. 2005; Heinrich and Wichmann 2004; Künzli et al. 2000; Nel 2005). Traffic-related pollutants such as particulate matter, nitrogen dioxide, and ozone are strong oxidants (D’Amato et al. 2005), leading to the production of reactive oxygen species (ROS). Oxidative stress triggers the inflammatory response and cytokine production (Kelly 2003; Nel 2005). It is plausible that genetic variants involved in inflammation and protection against ROS may influence the response to air pollutants. Polymorphisms in oxidative stress genes NAD(P)H:quinine oxidoreductase [*NQO1*, GeneID 1728 (Entrez Gene 2008)] and gluthatione *S*-transferases M1 [*GSTM1*, GeneID 2944 (Entrez Gene 2008)] and P1 [*GSTP1*, GeneID 2950 (Entrez Gene 2008)] have been associated with a decrease on pulmonary function (Bergamaschi et al. 2001; Romieu et al. 2004; Yang et al. 2005) and with increased allergic response (Gilliland et al. 2004, 2006), respiratory symptoms, and asthma (David et al. 2003; Lee et al. 2004; Li et al. 2006; Romieu et al. 2006) in response to air pollutants, including O_3_ and diesel exhaust particles (DEP). Variants in the proinflammatory cytokine gene tumor necrosis factor α [*TNFA*, GeneID 7124 (Entrez Gene 2008)] have been linked to decrease in pulmonary function (Winterton et al. 2001; Yang et al. 2005) and asthma and wheezing (Li et al. 2006) in relation to O_3_ and sulfur dioxide exposure. A recent study using nitrogen oxides (NO_x_) as an indicator for local traffic air pollution has reported interaction effects between *GSTP1* polymorphisms and NO_x_ on allergic sensitization to common allergens in children at 4 years of age. This interaction was more pronounced in carriers of *TNFA* −308 GA or AA genotype (Melén et al. 2008). Toll-like receptor 4 [*TLR4*, GeneID 7099 (Entrez Gene 2008)], implicated in innate immunity and endotoxin responsiveness, is also a candidate to be involved in air pollution susceptibility (Romieu et al. 2008; Saxon and Diaz-Sanchez 2005; Yang et al. 2008). In mouse models, lung hyperpermeability induced by O_3_ has been linked to the chromosome region containing the *TLR4* gene (Kleeberger et al. 2000, 2001). Another plausible candidate gene to modify the effects of air pollution is adrenergic receptor β2 [*ADRB2*, GeneID 154 (Entrez Gene 2008)]. It has been shown that polymorphisms in this gene interact with environmental tobacco smoke in children (Wang et al. 2008; Zhang et al. 2007) and cigarette smoke in adults (Joos et al. 2003; Litonjua et al. 2004; Wang et al. 2001). However, a previous study evaluating the combined effects of air pollutants and *ADRB2* in children did not report significant findings (Melén et al. 2008).

To date, evidence for gene–air pollution interactions on asthma have been reported only in children (Melén et al. 2008; Yang et al. 2008). Susceptibility to air pollution in early life may biologically differ from adulthood (Salam et al. 2008). Previous analysis on the European Community Respiratory Health Survey (ECRHS) reported that traffic-related pollution (using estimates of modeled NO_2_ exposure) was positively associated with new-onset asthma in adults (Jacquemin et al. 2009). The aim of the present study was to identify interactions between genes and traffic-related pollution on asthma incidence and prevalence in a large population-based cohort (ECRHS) of adults. For this purpose, we evaluated previously reported candidate genes that have a role in oxidative stress {*GSTM1*, glutathione *S*-transferase T1 [*GSTT1*, GeneID 2952 (Entrez Gene 2008)], *GSTP1*, and *NQO1*}, inflammatory response (*TNFA*), innate immunity (*TLR4*), and airway reactivity (*ADRB2*).

## Materials and Methods

### Study population

The methodology of ECRHS has been described elsewhere (Burney et al. 1994; ECRHS II Steering Committee 2002). Briefly, the ECRHS is a random population-based multicenter cohort of subjects 20–44 years of age at the time of recruitment (1990; ECRHS I) and then followed approximately 10 years later in ECRHS II (median length of follow-up, 8.9 years). In a first step of ECRHS I, random recruited subjects were contacted to complete a short questionnaire on respiratory symptoms. In a second step, a 20% random sample of participants was recontacted, completed a long questionnaire, and underwent some exams. A complementary sample of subjects with asthma symptoms at recruitment was also included in the study. Ethical approval was obtained for each center from the appropriate institutional ethics committee, and written consent was obtained from each participant.

For ECRHS II, modeled NO_2_ concentrations were assigned to a total of 5,470 participants in the 20 centers for which modeled air pollution was available. Genotyping was performed in 5,065 individuals from 19 centers, for which NO_2_ was measured for 2,920 participants in 13 centers: Sweden (Umeå and Uppsala), United Kingdom (Ipswich and Norwich), Spain (Albacete, Barcelona, Huelva, Galdakao, and Oviedo), Germany (Erfurt), France (Paris and Grenoble), and Belgium (Antwerp). For the present analysis, we excluded subjects from the symptomatic sample who did not report asthma in ECRHS II, leading to a final sample size of 2,577. No major differences were observed between the complete sample with estimated NO_2_, the subsample with DNA, and the subsample with both DNA and NO_2_ [see Supplemental Material, Table 1 (doi:10.1289/ehp.0900589.S1 via http://dx.doi.org)].

Subjects included in our analysis could be considered as mainly of European–Caucasian origin. The prevalence of asthma was based on a positive response to either of two questions in ECRHS II: attack of asthma during the last 12 months or current use of asthma medication. New-onset (incident) of asthma was defined as reporting asthma (either attack of asthma in preceding 12 months or current medication for asthma) in ECRHS II (follow-up) excluding people who reported asthma (same definition as above) or a history of asthma in ECRHS I (baseline). Persistent asthma was defined as reporting asthma (either attack of asthma in preceding 12 months or current medication for asthma) in both surveys.

We evaluated the robustness of our results with other definitions of asthma: ever asthma, defined as a positive response to “have you ever had asthma?” and physician-diagnosed asthma, defined as a positive response to “have you ever had asthma diagnosed by a doctor?” Participants also underwent a bronchial challenge with methacholine chloride administered by Mefar aerosol dosimeters (Mefar, Bovezzo, Italy). Bronchial hyperresponsiveness was defined as a 20% fall in forced expiratory volume in 1 sec (FEV_1_) from the highest FEV_1_ postdiluent during methacholine challenge with an accumulated dose of 1 mg (Burney et al. 1994; Chinn et al. 1997). Specific IgE levels to house dust mite, cat, timothy grass, and *Cladosporium herbarum* fungus were measured with the Pharmacia CAP system (Pharmacia Diagnostics, Uppsala, Sweden). Atopy was defined as sensitization (IgE levels > 0.35 kU/L) to any allergen.

### Modeled NO_2_ concentrations

NO_2_ has been widely used as a marker of local traffic-related air pollution (Emenius et al. 2003; Forastiere et al. 2006; Jacquemin et al. 2009; Modig et al. 2006; Morgenstern et al. 2008). NO_2_ measurements substantially differ within cities because they capture differences in exposure due to different proximities to traffic arteries. Details on modeling of NO_2_ concentrations are described in the Supplemental Material (doi:10.1289/ehp.0900589.S1). Briefly, as part of the Air Pollution Modelling for Support to Policy on Health and Environmental Risk in Europe project (APMoSPHERE 2007), 1-km- resolution emission maps were developed. The NO_x_ emission map was used as the basis for modeling NO_2_ concentrations using focal sum techniques in a global information system model. The NO_2_ at the place of residence for each subject was then obtained by intersecting the point locations of their residence with the air pollution map.

### DNA extraction and genotype characterization

Polymorphisms of the genes *GSTM1*, *GSTT1*, and *GSTP1* were selected according to functional evidence from existing literature. For the genes *NQO1*, *TNFA*, *TLR4*, and *ADRB2*, we selected tag single-nucleotide polymorphisms (SNPs) in the gene region, 10 kb upstream from the 5′ untranslated region (UTR), and 10 kb downstream from 3′ UTR. Polymorphisms are listed in Table 1.

DNA was extracted from blood cells for samples using a commercially available kit (Puregene, Gentra Inc., MN, USA). The DNA bank was built and maintained at Helmholtz Zentrum München in Germany. Genotyping was performed at the Centre for Genomic Regulation in the Barcelona Node of the Centro Nacional de Genotipado (2009) in Spain. *GSTM1* and *GSTT1* genotypes were determined using polymerase chain reaction method and *GSTP1* polymorphism by specific pyrosequencing assay. Genotyping for *NQO1*, *TNFA*, and *TLR4* polymorphisms was performed using the SNPlex platform (Applied Biosystems, Foster City, CA, USA). The average genotyping rate was 98%.

### Statistical analysis

The statistical analyses were performed using logistic regression implemented in the SNPassoc (version 1.5) (Gonzalez et al. 2007) package in R (version 2.6.1; R Foundation for Statistical Computing, Vienna, Austria). Generalized additive models (GAMs) were used to evaluate dose–response relationships with NO_2_. Logistic models and GAMs were adjusted for center, sex, age, environmental tobacco smoke, and smoking status. Multiplicative interactions were assessed using likelihood-ratio test comparing models with additive term and interaction term. Heterogeneity was evaluated using the Mantel–Haenszel method under fixed-effects model with the R library rmeta package (version 2.14). Logistic mixed-effects models allowed the evaluation of a random effect of the variable center. These models were also adjusted for the previously described covariates.

We tested deviations of genotype frequencies from Hardy–Weinberg equilibrium (HWE) (Wigginton et al. 2005) in the randomly selected population. To check the independence of the polymorphisms, we estimated the correlation (*R*^2^) and linkage disequilibrium coefficient (*D*′). Haplotypes were estimated using the haplo.em function from the haplo.stats package (version 1.3.8) (Schaid et al. 2002). Population stratification was assessed with the analysis of 26 unlinked markers [see Supplemental Material, Table 2 (doi:10.1289/ehp.0900589.S1)] using two different methods. First, the genomic control approach (Devlin and Roeder 1999) showed a minimal effect [inflation factor (λ) = 1.06]. Second, principal component analysis using the EIGENSTRAT method (version 1.01) (Price et al. 2006) showed no evidence of population stratification [see Supplemental Material, Figure 1 (doi:10.1289/ehp.0900589.S1)].

Additional information on material and methods can be found in the Supplemental Material (doi:10.1289/ehp.0900589.S1).

## Results

Table 2 lists population characteristics. The prevalence of current asthma in this sample was 12.7% (*n* = 327). Compared with subjects without asthma, asthmatics more often were women, were younger, reported less smoking, and had lower percentage of predicted lung function. Distribution of NO_2_ was similar to the previously reported for the whole population (Jacquemin et al. 2009) [see Supplemental Material, Table 3 (doi:10.1289/ehp.0900589.S1)]. The multivariate analysis of NO_2_ and prevalence of asthma indicates a small but not significant increase in asthma for each 10-μg/m^3^ increase in NO_2_ [odds ratio (OR) = 1.19; 95% confidence interval (CI), 0.97–1.47]. We observed departure from the HWE for *GSTP1* rs16951 (*p* < 0.01). Allele distribution by center was heterogeneous for *TNFA* rs2844484 and *TNFA* rs909253 variants (*p* < 0.01).

**Figure 1:**
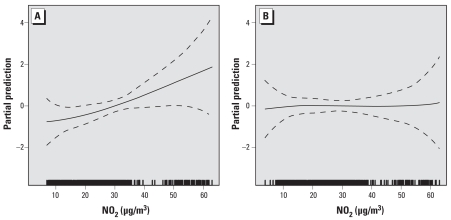
Spline graphics using GAM for the association between NO_2_ and current asthma, stratified by *NQO1* rs2917666 genotypes. (*A*) C/C carriers. (*B*) G/C or G/G carriers. Dashed lines indicate 95% CI.

For each genetic polymorphism, we evaluated the association between NO_2_ (per 10-μg/m^3^ increase) and asthma prevalence separately for carriers of the minor allele (either homozygous or heterozygous) and for the subjects homozygous for the major allele (Table 1). We found no statistically significant associations between NO_2_ and asthma for any of the genotypes of *GSTM1*, *GSTT1*, *GSTP1*, *TLR4*, and *ADRB2* genes. The association between prevalence of asthma and NO_2_ was significant for subjects homozygous for the major allele of *NQO1* rs2917666 (OR = 1.54; 95% CI, 1.10–2.24) and for *TNFA* rs2844484 (OR = 2.02; 95% CI, 1.30–3.27). A test for interaction between these polymorphisms and NO_2_ was significant only for *NQO1* rs2917666 (*p*-value for interaction = 0.02). Analysis using GAMs (Figure 1) indicated a significant linear increase in prevalence for homozygotes of the most prevalent alleles in *NQO1* rs2917666 (*p* = 0.02) and no increase for the G/C and G/G genotypes.

We further performed the haplotype analysis for the three SNPs of *NQO1* (rs10517, rs1800566, and rs2917666) in relation to NO_2_ exposure and prevalence of asthma (Table 3). In this analysis, we provide estimates for exposure among subjects within combinations of SNPs. Linkage disequilibrium was weak between some of the *NQO1* SNPs (*D*′ = 0.97, *r*^2^ = 0.03, and *p* < 0.01 between rs10517 and rs1800566; *D*′ = 0.86, *r*^2^ = 0.21, and *p* < 0.01 between rs10517 and rs2917666; *D*′ = 0.99, *r*^2^ = 0.53, and *p* < 0.01 between rs1800566 and rs2917666). The association between asthma prevalence and NO_2_ was significant for the most prevalent haplotype, composed of the three major alleles of each SNP (OR = 1.23; 95% CI, 1.03–1.48). This was the only haplotype containing the C allele of rs2917666, which showed a significant interaction with NO_2_ in the single SNP analysis. We observed no significant associations among carriers of the other haplotypes (Table 3).

We also analyzed longitudinal data to evaluate the effect of NO_2_ on new-onset asthma. We observed a significant association between new-onset asthma and NO_2_ levels for the 120 subjects who developed asthma during the follow-up period (OR = 1.52; 95% CI, 1.09–2.16). Subjects homozygous for the *NQO1* rs291766 C allele were at greater risk (*p*-value for interaction = 0.04) for developing asthma (OR = 2.02; 95% CI, 1.16–3.73) compared with those with CG/GG genotypes (OR = 1.26; 95% CI, 0.83–1.99).

We restricted the analysis to those subjects who lived in the same home during follow-up (*n* = 1,348) to reduce exposure misclassification. Compared with subjects who changed homes, this group had an increased risk for main effects of exposure to NO_2_ on asthma prevalence (movers: OR = 1.64; 95% CI, 1.08–2.53; nonmovers: OR = 1.02; 95% CI, 0.80–1.31; *p*-value for interaction = 0.03), whereas the effect on new-onset asthma was not different between movers and nonmovers (movers: OR = 1.48; 95% CI, 0.83–2.74; nonmovers: OR =1.59; 95% CI, 1.05–2.52; *p*-value for interaction = 0.81). CC carriers of *NQO1* rs2917666 living in the same home during follow-up had an increased risk for prevalence (OR = 2.42; 95% CI, 1.19–5.24) and new-onset asthma (OR = 2.89; 95% CI, 1.02–9.46).

In a sensitivity analysis, we also evaluated our findings of interaction between *NQO1* rs2917666 and NO_2_ for other asthma definitions and asthma-related phenotypes [see Supplemental Material, Table 4 (doi:10.1289/ehp.0900589.S1)]. Interaction was significant for ever asthma (*p* = 0.006), physician-diagnosed asthma (*p* = 0.01), and asthmatics showing bronchial hyperresponsiveness (*p* = 0.02), whereas interactions were not significant for persistent asthma (*p* = 0.12) or atopy (*p* = 0.46). Stratification by atopic status showed that interaction between NO_2_ and *NQO1* rs2917666 was more pronounced among nonatopic carriers of *NQO1* rs2917666 C/C (OR = 2.38; 95% CI, 1.13–5.68; *p*-value for interaction = 0.01) compared with atopic subjects (OR = 1.24; 95% CI, 0.84–1.91; *p*-value for interaction = 0.45).

When stratified by sex [see Supplemental Material, Table 5 (doi:10.1289/ehp.0900589.S1)], interaction between NO_2_ and rs2917666 was significant only among females (C/C: OR = 1.81; 95% CI, 1.13–3.09; *p*-value for interaction = 0.03) and not in males (C/C: OR = 1.39; 95% CI, 0.82–2.46; *p*-value for interaction = 0.24). However, heterogeneity by gender was not significant (*p* = 0.71). We observed no significant differences in the effect of NO_2_ and *NQO1* rs2917666 on current asthma by center (*p*-value for heterogeneity = 0.51). In addition, after including the random effects of center in a generalized mixed model, the interaction of NO_2_ and *NQO1* rs2917666 was still significant for the prevalence of asthma (*p*-value for interaction = 0.02). Heterogeneity was not significant among different socioeconomic strata based on occupation (*p*-value for heterogeneity = 0.28). The exclusion of non-randomly selected symptomatic subjects in the second step of ECRHS I did not modify the effects observed in the whole sample (*p*-value for heterogeneity = 0.36)

## Discussion

We evaluated the effect of genes involved in oxidative stress pathways (*GSTM1*, *GSTT1*, *GSTP1*, and *NQO1*), inflammatory response (*TNFA*), immunologic response (*TLR4*), and airway reactivity (*ADRB2*) on the association of traffic-related air pollution (using estimates of modeled NO_2_ exposure) and adult asthma in a large, multicenter population-based cohort. We observed stronger associations between NO_2_ concentrations and both prevalent and new-onset asthma among subjects homozygous for the most common allele of *NQO1* compared with carriers of *NQO1* variants. Although several studies have evaluated similar gene–environment interactions in children (Bergamaschi et al. 2001; David et al. 2003; Lee et al. 2004; Li et al. 2006; Melén et al. 2008; Romieu et al. 2004, 2006) and in experimental settings in adults (Corradi et al. 2002; Gilliland et al. 2004, 2006; Winterton et al. 2001; Yang et al. 2005), this is the first study examining the interaction of genetic variation and long-term air pollution on asthma in adults.

Similar to other studies, we used NO_2_ level as a marker of traffic-related air pollution (Emenius et al. 2003; Forastiere et al. 2006; Jacquemin et al. 2009; Melén et al. 2008; Modig et al. 2006; Morgenstern et al. 2008). Thus, the observed associations and interactions may be mediated by other ambient air pollutants, which are highly spatially correlated with NO_2_. However, NO_2_ is a strong oxidant per se, with a range of well-known adverse effects (Forastiere et al. 2006; Janssen-Heininger et al. 2002), and NO_2_ either alone or combined with other pollutants may contribute to the observed effects (Forastiere et al. 2006).

In our study, we found a nonsignificant reduction of the NO_2_ effects among carriers of at least one *NQO1* Ser187 allele. The polymorphism that was associated with the most significant *p*-value in our study (rs2917666) is not known to be functional but is located in the 5′ upstream region of the gene. This region contains elements essential for the expression and induction of *NQO1*, such as the antioxidant response element that is required for *NQO1* transcription in response to oxidative stress (Jaiswal 2000; Nioi and Hayes 2004). The three SNPs in the *NQO1* gene were in relatively weak linkage disequilibrium with the highest *r*^2^ (0.53) found for the functional Pro187Ser (rs1800566) and rs2917666. Furthermore, the association between asthma and modeled NO_2_ was significant for the most prevalent haplotype that contained the C allele of the rs2917666, which showed a significant interaction with NO_2_ in the single SNP analysis. A few studies have evaluated the role of *NQO1* in relation to exposure to O_3_ [reviewed by Yang et al. (2008)] and have shown that the Pro187Ser (rs1800566) polymorphism was protective in response to O_3_ when *GSTM1* was present (Bergamaschi et al. 2001; Corradi et al. 2002; David et al. 2003). Susceptibility variants on oxidative stress genes *GSTM1* and *GSTP1* have been associated with an increased effect of air pollution and specific pollutants (Gilliland et al. 2004; Lee et al. 2004; Li et al. 2006; Melén et al. 2008; Romieu et al. 2004, 2006). In this study, we did not observe significant associations of *GSTM1* or *GSTP1* polymorphisms with asthma either alone or in combination with *NQO1* SNPs. Lack of consistence with previous analyses could be related to the heterogeneity of effects in adults compared with children (Salam et al. 2008).

Our findings on *NQO1* reinforce the role of antioxidant system in response to air pollution (Kelly 2003; Romieu et al. 2008). An *in vitro* approach proposed a hierarchical model to explain the dose-dependent response to oxidant chemicals in DEP (Xiao et al. 2003) that will probably extend to gaseous pollutants like NO_2_ (Saxon and Diaz-Sanchez 2005). With low exposure, the formation of ROS activates the transcription of genes involved in antioxidant responses, such as phase II enzymes (e.g., *NQO1* and GST genes). Higher exposure activates the transcription of nuclear factor-κB and activator protein-1, resulting in increased expression of proinflammatory cytokines (e.g., TNF-α), leading to additional generation of ROS (Romieu et al. 2008; Saxon and Diaz-Sanchez 2005).

The ECRHS is a large population-based international cohort that overcomes limitations of studies done in selected populations. The main limitations of this analysis include limited statistical power to detect interactions, some exposure misclassification, and heterogeneity and potential confounding concerning environmental exposures and genetic variation.

Statistical power to detect interactions was relatively low (Garcia-Closas et al. 1999). False-positive results have been shown to be frequent in studies on genetic variation and gene–environment interactions (Clayton and McKeigue 2001), and for these reason these results should be interpreted with caution. In this study we did not perform correction for multiple comparisons. However, traditional methods based on Bonferroni are overconservative because polymorphisms within a gene are not completely independent due to linkage disequilibrium. In addition, this correction may be acceptable when searching for significant associations without preestablished hypotheses, but we selected genes in this analysis on the basis of strong prior evidence.

Strengths and limitations of the NO_2_ exposure assessment have been discussed previously (Jacquemin et al. 2009). The individual assignment of exposure to NO_2_ should result in a reduction of exposure misclassification. We evaluated NO_2_ exposure by geocoding home addresses of ECRHS participants and assigned ambient modeled NO_2_ concentration derived from the APMoSPHERE map to each subject. The year of modeled NO_2_ (2001) was concordant with the years of the administration of the ECRHS II questionnaire (1999–2002). However, the spatial scale of the APMoSPHERE model was relatively broad (1 × 1 km), and the model did not include monitors placed close to traffic. Thus, spatial and temporal contrasts in exposure due to very local emissions and dispersion patterns, such as those occurring in street canyons are unlikely to be captured. NO_2_ does also capture part of that space but as a secondary gas is certainly more homogeneously distributed than, for example, ultrafine tail pipe particles. The misclassification is random in nature so, if anything, some bias toward the null may be expected. If those local peak concentrations were particularly relevant sources of exposure to oxidants, the APMoSPHERE-based results would likely underestimate true effects and interactions.

Because of the lack of repeated measurements during follow-up, exposure was assigned only to residences in ECRHS II. Although levels of air pollution did not remain constant during the follow-up period, the ranking in the spatial distribution of traffic-related pollutants is likely to remain similar. Exposure misclassification is thus particularly large among those who moved after ECRHS I, possibly explaining the smaller effects observed among movers (Beelen et al. 2008; Gotschi et al. 2008a).

Levels of air pollution and prevalence of asthma varied substantially across centers in ECRHS, showing a south–north gradient (Gotschi et al. 2008b; Jacquemin et al. 2009; Sunyer et al. 2004). Median levels of modeled NO_2_ varied from 12 μg/m^3^ in Umeå to more than 50 μg/m^3^ in Barcelona and Paris (Jacquemin et al. 2009). Variables correlated with center, such as pollution composition, climatic factors, and diet, determine the individual response to air pollution (D’Amato et al. 2005). All results were adjusted by center, and random-effects meta-analysis suggested that NO_2_ estimates and interactions with the genes were not center specific, although we acknowledge the limited power to detect this heterogeneity. In addition, recent analyses of population stratification among Europeans found correspondence between genetic variation and geographic distances, although levels of genetic diversity were low (Heath et al. 2008). Previous analyses in the ECRHS have shown little evidence of population stratification (Castro-Giner et al. 2008). However, this was based on an insufficient set of markers.

An important source of NO_2_ exposure in the general population originates from the use of gas cookers (D’Amato et al. 2005). Several studies reported the association of indoor exposures with NO_2_ outcomes and respiratory or allergic outcomes (Bernstein et al. 2008). In our data, outdoor NO_2_ was not correlated with gas cooking, and adjustment for cooking with gas did not affect the observed effects of outdoor NO_2_ and its interaction with the *NQO1* polymorphism.

Differences were observed by sex, with females showing an increase in risk. However, the sample sizes of specific strata are smaller than the total population, and interpretation of these results should be done with caution. Our findings, if corroborated by others, may have significant public health implications because we identified a large group of susceptible subjects defined by the genetic makeup for whom the effect of modeled NO_2_-related air pollution on asthma was substantial. The affected subgroup was large, with a 46% prevalence of the C/C genotype for *NQO1* rs2917666. Moreover, the number of people exposed to traffic-related pollution on a regular basis is large and as a consequence the burden of asthma related to ambient air pollution may be large not only in children, as previously documented, but also in adults.

## Conclusions

Findings from this study suggest that genetic polymorphisms in the *NQO1* gene are associated with susceptibility to asthma in adults among those exposed to traffic-related air pollution. This result points to the importance of antioxidant pathways in the effects of air pollution on asthma.

## Figures and Tables

**Table 1:** Association of NO_2_ (per 10 μg/m^3^) and asthma prevalence according to polymorphisms in ECRHS II.

Gene	Polymorphism	Minor allele frequency	Coding change	Variant	No. asthmatics (%)	OR (95% CI)
*GSTM1*	Deletion	49.0	—	Present	134 (12.0)	1.10 (0.8–1.54)
				Null	152 (13.2)	1.06 (0.78–1.46)

*GSTT1*	Deletion	20.0	—	Present	217 (12.0)	0.98 (0.78–1.26)
				Null	69 (15.2)	1.69 (0.97–3.07)

*GSTP1*	rs1695	32.0	Exon 5 Ile105Val	A/A	104 (11.2)	1.34 (0.97–1.93)
				A/G+G/G	211 (14.1)	1.10 (0.84–1.46)

*NQO1*	rs10517	11.9	3′ UTR region	C/C	131 (14.1)	1.20 (0.96–1.53)
				C/T+T/T	193 (11.8)	1.11 (0.71–1.82)

*NQO1*	rs1800566	20.2	Exon 6 Pro187Ser	C/C	222 (12.7)	1.36 (1.03–1.84)
				C/T+T/T	102 (12.5)	1.06 (0.79–1.45)

*NQO1*	rs2917666	32.2	3′ downstream	C/C	163 (12.8)	1.54 (1.10–2.24)
				C/G+G/G	164 (12.7)	1.01 (0.79–1.33)

*TLR4*	rs10759930	40.1	5′ upstream	C/C	143 (13.5)	1.13 (0.80–1.65)
				C/T+T/T	163 (12.3)	1.21 (0.95–1.58)

*TLR4*	rs11536889	14.3	3′ UTR region	G/G	255 (12.9)	1.22 (0.96–1.58)
				G/C+C/C	72 (12.3)	1.24 (0.83–1.98)

*TLR4*	rs1554973	23.7	3′ downstream	T/T	207 (12.5)	1.19 (0.91–1.58)
				T/C+C/C	119 (13.0)	1.19 (0.87–1.66)

*TLR4*	rs1927914	32.5	5′ UTR region	T/T	155 (12.9)	1.20 (0.89–1.65)
				T/C+C/C	171 (12.5)	1.18 (0.90–1.58)

*TLR4*	rs2737191	27.3	5′ upstream	A/A	115 (12.4)	1.28 (0.96–1.73)
				A/G+G/G	212 (13.0)	1.10 (0.82–1.49)

*TNFA*	rs1800629	16.1	5′ upstream	G/G	207 (12.0)	1.27 (0.97–1.69)
				G/A+A/A	69 (11.1)	1.22 (0.86–1.80)

*TNFA*	rs2844484	41.2	5′ upstream	C/C	196 (13.1)	2.02 (1.30–3.27)
				C/T+T/T	131 (12.2)	1.02 (0.81–1.30)

*TNFA*	rs909253	31.2	Intron 3 of LTA	A/A	146 (12.4)	1.29 (0.96–1.76)
				A/G+G/G	180 (13.0)	1.14 (0.86–1.54)

*ADRB2*	rs1042713	38.8	Exon 1 Arg16Gly	G/G	166 (12.3)	1.03 (0.73–1.50)
				G/A+A/A	160 (13.2)	1.18 (0.91–1.56)

*ADRB2*	rs1042714	40.0	Exon 1 Gln27Glu	C/C	197 (12.0)	1.21 (0.88–1.70)
				C/G+G/G	102 (14.9)	1.20 (0.92–1.60)

*ADRB2*	rs1042718	17.7	Exon 1 synonymous	C/C	115 (12.3)	1.21 (0.94–1.57)
				C/A+A/A	212 (12.9)	1.20 (0.79–1.65)

*ADRB2*	rs1042719	30.4	Exon 1 synonymous	G/G	147 (12.0)	1.23 (0.92–1.68)
				G/C+C/C	180 (13.4)	1.19 (0.89–1.63)

**Table 2:** Population characteristics at follow-up (ECRHS II) for subjects with both DNA and assigned levels of NO_2_.

	All	No asthma	Asthma	*p*-Value
Subjects (no.)	2,577	2,250	327	—
Females [no. (%)]	1,345 (52.2)	1,154 (51.3)	191 (58.4)	0.02
Age [years (mean ± SD)]	43.03 ± 7.3	43.2 ± 7.2	41.83 ± 7.2	0.001
Smoking status [no. (%)]				
Never	1,130 (44)	969 (43.1)	161 (49.2)	—
Former	714 (27.8)	624 (27.8)	90 (27.5)	—
Current	729 (28.3)	653 (29.1)	76 (23.2)	0.05
Same house during follow-up [no. (%)]	1,348 (52.3)	1,192 (53.0)	156 (47.7)	0.08
Percent predicted FEV_1_ (mean ± SD)	106.81 ± 15.2	108.86 ± 13.9	95.58 ± 18.2	< 0.001

**Table 3:** Association of NO_2_ per 10-μg/m^3^ increase and asthma prevalence stratified by *NQO1* and haplotypes in ECRHS II.

Haplotype	Allele			Prevalence	OR (95% CI)	*p*-Value	*p*-Value for interaction
	rs10517	rs1800566	rs2917666				
1	C	C	C	0.66	1.23 (1.03–1.48)	0.03	Reference
2	C	T	G	0.21	1.28 (0.90–1.89)	0.20	0.50
3	T	C	G	0.11	1.11 (0.72–1.81)	0.66	0.73
